# Chronic Abscess

**Published:** 1896-06

**Authors:** M. L. Rhein

**Affiliations:** New York.


ARTICLE II.

### CHRONIC ABSCESS.

DR. M. L. RHEIN, NEW YORK.

The same principles that apply to the treatment of the disease in the acute form hold good here, except that, if possible, a more thorough form of procedure is necessary. In the treatment of chronic abscess, the first requisite (as it should be in all diseases) is the making of a thorough diagnosis. By that is meant (in this disease) so thorough an examination of the gums, roots, and all the adjacent parts, by means of probes, electric light, touch, etc., that a very clear understanding is obtained of the pathological conditions from an anatomical standpoint.

Having these conditions clearly outlined, it is folly to pursue any preliminary treatment of such a tooth. Our object should be to eradicate as rapidly as possible every vestige of diseased tissue, and then allow nature to close the wound.

The first step is necessarily the thorough exploration and cleansing of the canals to their very end. No obstacle in the shape of prior fillings or treatment should be allowed to interfere with this operation. Having thoroughly accomplished this object, no further benefit can be obtained by internal treatment; so that it is generally wise to fill such a canal at the first sitting, if it has been cleansed to its very ends.

In all forms of chronic abscess, there is present caries of the outer periphery of the end of the root, as well as of the alveolar plate in which the root is imbedded. As long as any necrosed bone is allowed to remain, it will be impossible to effect a permanent cure. Surgical interference is logically the only resource at our command.

The greatest criticism that can be passed on us as a profession, in treating diseased conditions of this nature, is that by the minute character of the daily work in which we are engaged, when we come to operate on diseased tissue we have a strong inclination to err by being too conservative in the scope of our operative interference.

If we desire to cure chronic abscess, we must throw aside all that delicacy of touch which is our daily pride in ordinary dental operations. That we may leave behind no diseased tissue we must follow the example of the general surgeon, and enter freely into the zone of healthy bone and tooth structure.

The incision should be made sufficiently large, so that by means of sharp burs in the dental engine we may readily remove an ample sufficiency of the bone in the diseased tract. The most efficient method is to take the ordinary fissure-drill in the dental engine (the sinus having been enlarged by trephines or otherwise), pass it down the tract along the side of the diseased root low enough toward the crown of the tooth to be certain that you have passed into the healthy tissue, and there, by means of the drill, amputate that portion of the root which is necrosed.

Sometimes this is surprisingly easy, while in others it is difficult. What ever cbstacles may have to be surmounted, if the tooth is one that is to be preserved in a healthy condition, this is the only safe method. Some times it will be necessary to place the patient under the influence of a general anæsthetic, especially where it is necessary to bur away the diseased portion of the alveolar process, that the end of the amputated root may be removed.

A common mistake of dentists is to look on this operation as something beyond their scope, and to fear the trending on what they might term dangerour surgical ground.

It would surprise many who are unacquainted with this operation to discover what a simple affair it becomes after they have amputated the ends of a few roots. No one is better constituted to perform such an operation than a good dentist, and in no place are there such facilities for properly performing the operation as are found in a properly-equipped dental office.

The most difficult forms that require surgical interference are those which have no external opening, but from time to time visibly demonstrate that something serious is amiss. I refer specially to those known as "blind abscesses," The internal treatment of the roots having been performed as described, it is necessary to make an artificial sinus directly to that portion of the root where we wish to sever it from the main body of the tooth. If no general anæsthetic be administered, the gum at this point should have injected into it about ten minims of a 4 per cent freshly-prepared solution of the hydrochlorate of cocain. A trephine rapidly revolved in the engine will very quickly and almost painlessly effect the desired result. This newly-formed sinus can then be enlarged most readily by means of a crucial incision made by a heavy scalpel, and the root reached by means of a sharp croes-cut inverted cone bur. The remainder of the operation is similar to what has been described.

Another method of producing an enlargement of the sinus, either if it be freshly made or an old tract, is to pack it solidly with a tent of cotton dipped in aromatic sulphuric acid, allowed to remain there for twenty-four hours. This is by no means as desirable as immediate surgical interference, as it is not only productive of considerable pain caused by the swelling of the tent, but the destructive effect of the aromatic sulfuric acid on the soft tissues is very detrimental to a prompt closure of the wound.

In teeth with more than one root the amputation of the entire root affected by the disease is often to be preferred to the risk of leaving behind some necrosed tooth-substance. The end of the stump can be nicely smoothed and hermetically closed with an amalgam filling, which at a later period should be polished.

In pyorrhea of all kinds, we meet with many molars where the roots have become entirely denuded. This is especially true of the palatal roots of upper molars, and the phorrheal condition has for a long time been complicated with abscess. To remove the palatal root of such a tooth has become frequent with me. I have yet to see the first case where the operation has not succeeded in permanently tightening these very loose teeth.

After the roots of such teeth are filled, the removal of such a palatal root is wonderfully simple. The parts are all exposed, and it takes but a few revolutions of the bur to amputate close to the crown, filling the end of the stump with amalgam, and later polishing the same.

Summarizing the treatment of chronic abscess, the same course should be pursued in the canals of the teeth as in the acute stage, at the first sitting, if possible, simply for the purpose of hermetically sealing the interior of the tooth. The diseased tissue is then to be entirely eradicated by surgical interference through an opening in the gum over the root, and if this be effectively accomplished, the abscess is radically cured.—*Cosmos.*

